# Olympic Snow Sports: Current Insights and Future Directions for Milano Cortina 2026 and Beyond

**DOI:** 10.1111/sms.70222

**Published:** 2026-02-07

**Authors:** Chiara Zoppirolli, Alessandro Fornasiero, Jörg Spörri, Thomas Losnegard, Ola Anker Elfmark, Øyvind Sandbakk, H.‐C. Holmberg

**Affiliations:** ^1^ CeRiSM (Research Centre of Mountain Sport and Health) University of Verona Rovereto Italy; ^2^ Department of Neuroscience, Biomedicine and Movement University of Verona Verona Italy; ^3^ Sports Medical Research Group, Department of Orthopaedics Balgrist University Hospital, University of Zurich Zurich Switzerland; ^4^ University Centre for Prevention and Sports Medicine Balgrist University Hospital, University of Zurich Zurich Switzerland; ^5^ Department of Physical Performance Norwegian Oool of Sport Sciences Oslo Norway; ^6^ Centre for Elite Sports Research, Department of Neuromedicine and Movement Science Norwegian University of Science and Technology Trondheim Norway; ^7^ School of Sport Science UiT The Arctic University of Norway Tromsø Norway; ^8^ Division of Machine Elements Luleå University of Technology Luleå Sweden; ^9^ Department of Physiology and Pharmacology, Biomedicum C5 Karolinska Institutet Stockholm Sweden; ^10^ School of Kinesiology University of British Columbia Vancouver British Columbia Canada

**Keywords:** athlete development, biomechanics, elite athletes, performance determinants, physiology, sex differences, training characteristics, Winter Olympic Games

## Abstract

As the Milano Cortina 2026 Winter Olympic Games approach, a comprehensive understanding of performance determinants across Olympic snow sports is increasingly important to further evolve training and performance. However, the scientific literature remains unevenly distributed, with well‐established knowledge in cross‐country skiing, biathlon, and alpine skiing, and limited data in disciplines such as ski mountaineering, freestyle skiing, snowboarding, ski jumping, and Nordic combined. This narrative review synthesizes current evidence to (1) identify key performance‐determining factors, (2) describe discipline‐specific training characteristics, and (3) highlight critical knowledge gaps. Regarding performance determinants, Olympic snow sports can be broadly categorized into endurance‐dominant disciplines (e.g., cross‐country skiing, biathlon, ski mountaineering), which rely on high aerobic capacity and movement efficiency, and the gravity and technical disciplines (e.g., alpine skiing, freestyle skiing, snowboarding, ski jumping), which emphasize neuromuscular power and technical precision. Nordic combined represents a hybrid of these categories. In terms of training characteristics, elite athletes' training models reflect sport‐specific demands through tailored combinations of endurance, strength–power, technical, tactical, and psychological preparation. Finally, regarding knowledge gaps, sex‐specific analyses of physiological profiles, biomechanics, and training responses remain scarce, particularly in gravity and technical sports. Furthermore, standardized documentation of training structure, integration of on‐snow monitoring technologies, and research on energy availability remain underdeveloped. Addressing these gaps through holistic, multidisciplinary research is essential to develop individualized, sex‐informed, and evidence‐based frameworks that support athlete development and performance optimization in the lead‐up to Milano Cortina 2026 and future Olympic cycles.

## 1 Introduction

**FIGURE 1:**
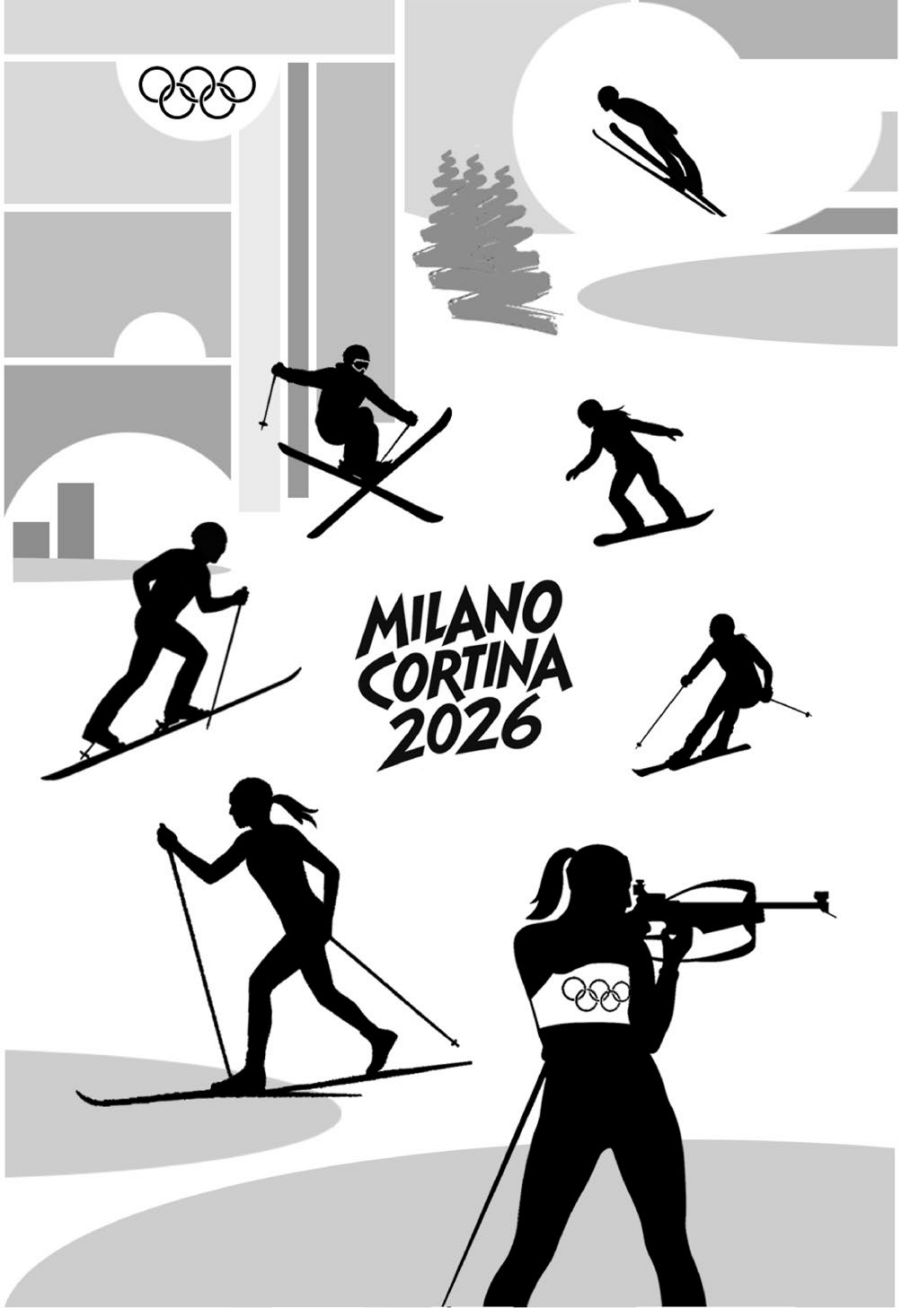
Artistic illustration of the Milano–Cortina 2026 Winter Olympic Games.

The Milano Cortina 2026 Winter Olympic Games will mark over a century since the inaugural Winter Olympics in Chamonix in 1924 (Figure 1). Since then, the Olympic snow‐sport program has evolved from a core of Nordic disciplines—cross‐country skiing, ski jumping, and Nordic combined (all debuted in 1924)—to encompass a diverse array of events, including alpine skiing (1936), biathlon (1960), freestyle skiing (1992), snowboarding (1998), and ski mountaineering (2026). While many of these disciplines initially featured only men's events, female competitions were introduced progressively (e.g., women's cross‐country skiing added in 1952, women's biathlon in 1992); however, Nordic Combined remains the only Olympic snow sport without a women's event for 2026. This evolution has broadened the spectrum of performance demands, ranging from sustained endurance efforts to high‐speed, acrobatic, and technically complex maneuvers.

Because of this diversification, the scientific understanding of Olympic snow sports remains uneven. Bibliometric data indicate that the literature is heavily concentrated in the traditional Nordic and Alpine disciplines (i.e., cross‐country skiing and alpine skiing), whereas disciplines with a shorter Olympic history—such as freestyle skiing, snowboarding, and ski mountaineering—remain comparatively under‐researched [1]. Moreover, sex‐specific analyses are limited across many disciplines, particularly in gravity and technical sports, where female athletes remain underrepresented in performance and training studies.

**FIGURE 2:**
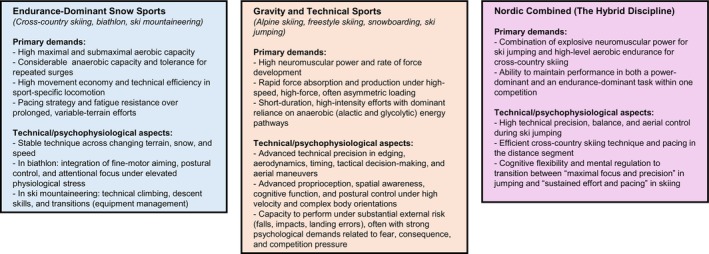
Conceptual taxonomy of Olympic snow sports based on primary performance demands. The framework positions sports along a continuum from endurance‐dominant, cyclic whole‐body exercise (cross‐country skiing, biathlon, ski mountaineering) to gravity and technical sports (alpine skiing, freestyle skiing, snowboarding, ski jumping). Nordic Combined represents a distinct hybrid discipline, necessitating the concurrent application of explosive neuromuscular power (ski jumping) and high‐level aerobic endurance (cross‐country skiing). Text boxes delineate the predominant physiological, technical, and psychophysiological characteristics of each category, emphasizing the disparate skill sets required for elite performance.

To provide a structured synthesis of the current evidence base, we categorize Olympic snow sports into two broad groups based on their primary performance demands: (1) *Endurance‐Dominant Snow Sports* (cross‐country skiing, biathlon, and ski mountaineering), which require high aerobic and anaerobic capacity, movement efficiency, and fatigue resistance in whole‐body exercise modes (with the critical addition of marksmanship in biathlon); and (2) *Gravity and Technical Sports* (alpine skiing, freestyle skiing, snowboarding, and ski jumping), which emphasize neuromuscular power, technical precision, tactical decision‐making, and the ability to perform under high‐speed, high‐force, and high‐risk conditions. Notably, Nordic combined spans both categories, requiring athletes to integrate explosive power and technical skill in ski jumping with endurance and skiing efficiency in cross‐country skiing (Figure 2).

The imminent Milano Cortina Games provide a timely opportunity to synthesize scientific knowledge across these disciplines. This review focuses exclusively on Olympic snow sports; companion manuscripts in this topical issue span the wider landscape of Winter Olympic performance, covering complementary disciplines and thematic areas. Of particular relevance to the determinants discussed here, we refer readers to in‐depth analyses of physiological limitations, altitude training, and endurance models [2, 3]; female athlete physiology, health, and energy availability [4]; pacing strategies and drafting [5]; carbohydrate fueling [6]; and sports technology, including equipment and GNSS [7].

Consequently, the primary aims of this narrative review are to (1) identify key performance‐determining factors, (2) describe discipline‐specific training characteristics, and (3) highlight current knowledge gaps. These insights are intended to provide a scientific update and inform athlete development and performance optimization strategies for the 2026 Olympic cycle and beyond.

## 2 Methodological Approach

This article is a narrative review guided by the SANRA (Scale for the Assessment of Narrative Review Articles) criteria [8]. We searched PubMed/MEDLINE, Web of Science, and Scopus from inception to November 24, 2025, supplemented by backward and forward citation chaining. The search terms included specific discipline names combined with keywords related to physiology, biomechanics, performance, and training. Eligible studies included peer‐reviewed original research describing physical or technical characteristics of elite (national‐ to world‐class; Tiers 3–5) Winter Olympic athletes [9]. Where peer‐reviewed data were unavailable, we supplemented these findings with official regulations, competition results, and technical reports from international federations (e.g., FIS, IBU). We synthesized these findings to address the specific aims of the review.

## 3 Performance Outcomes and Training Characteristics Across Olympic Snow Sports

Consistent with the framework established in the Introduction, this section first synthesizes the evidence for endurance‐dominant sports, followed by an analysis of the gravity‐ and technical‐oriented disciplines. We conclude with the hybrid discipline of Nordic combined, examining its unique integration of these conflicting physiological demands.

### 3.1 Endurance‐Dominant Snow Sports

Endurance‐dominant snow sports require sustained, whole‐body locomotion characterized by exceptionally high metabolic turnover. While maximal aerobic power remains the cornerstone of performance, success in modern Olympic events is increasingly dependent on anaerobic capacity, upper‐body strength, and movement efficiency to manage high‐intensity tactical elements (e.g., overtaking, start/stop dynamics, final sprints), and to adapt to variable terrain profiles and friction conditions. Consequently, athletes in these disciplines sustain the highest annual training volumes of all Winter Olympic sports to optimize cardiovascular, muscular, and hematological adaptations. Although cross‐country skiing, biathlon, and ski mountaineering share this common physiological foundation, performance is further distinguished by discipline‐specific constraints (Table 1)—most notably, the hilly and undulating course profiles in cross‐country skiing and biathlon, the fine motor control required for marksmanship in biathlon and the substantial vertical gain, technical descents, and rapid and equipment transitions characteristic of ski mountaineering.

**TABLE 1:** Overview of event characteristics, format regulations, and performance metrics for endurance‐dominant snow sports at the Milano–Cortina 2026 Olympic Winter Games.

Sport	Event	Category	Format	Performance metrics	M	W	Sex gap (%)
Cross‐Country Skiing (12)	Sprint	M&W	1.2 km (C); Q (IS/TT); finals heats (QF/SF/Final)	Time (mm:ss)	≈2:30–3:00	≈2:50–3:15	≈13%–17%
	Team Sprint	M&W	1.2 km (F); Q (IS; 1 lap/ath; times summed); Final (relay; 6 laps; 3/ath)	Aggr. time (min)	≈15–18	≈16–19	≈11%–14%
	10 km	M&W	(F); IS	Time (min)	≈20–23	≈22–25	≈10%–14%
	Skiathlon	M&W	20 km; MS; 10 km (C) + 10 km (F)	Time (min)	≈44–50	≈48–54	≈10%–12%
	50 km	M&W	(C); MS; feed zones	Time (min)	≈115–125	≈135–145	≈10%–12%
	Relay	M&W	4 × 7.5 km; MS; legs 1–2 (C), 3–4 (F)	Aggr. time (min)	≈65–75	≈75–85	≈10%–12%
Biathlon (11)	Individual	M&W	20 km (M)/15 km (W); 4 SB (P–S–P–S)	Time (min)	≈42–45	≈37–39	≈11%^a^
	Sprint	M&W	10 km (M)/7.5 km (W); 2 SB (P–S)	Time (min)	≈21–23	≈17–19	≈12%^a^
	Pursuit	M&W	12.5 km (M)/10 km (W); PS; 4 SB (P–P–S–S)	Time (min)	≈27–28	≈24–26	≈12%^a^
	Mass Start	M&W	15 km (M)/12.5 km (W); MS; 4 SB (P–P–S–S)	Time (min)	≈32–35	≈29–33	≈12%^a^
	Relay	M&W	4 × 7.5 km (M)/4 × 6 km (W); MS; 2 SB/leg (P–S)	Aggr. time (min)	≈65–75	≈60–70	≈14%^a^
	Mixed Relay	M + W	4 × 6 km; MS; W–W–M–M; 2 SB/leg (P–S)	Average leg time (min)	≈12–14	≈15–17	≈15%^a^
Ski Mountaineering (3)	Sprint	M&W	max 70 m d+; Q (TT) + KO (QF/SF/Final)	Ind. time (mm:ss)^b^	≈2:30–3:15	≈3:00–3:45	≈17%–25%
	Mixed Relay	M + W	max 150 m d+; Q (TT) + MS (Final); 4 laps (2/ath); W–M–W–M	Average leg time (mm:ss)^b^	≈6:30–10:00	≈7:30–11:30	≈14%–21%

*Note:* Number of medal events in parentheses. Sex Gap (%): Estimated percentage difference between men and women in the reported performance metric (time or speed). Times are reported in minutes (min) unless otherwise indicated (mm:ss).

Abbreviations: Aggr., aggregate (total) time; ath, athlete; C, classic technique; d+, vertical gain; F, free technique (ski kating); Ind., individual time; IS, interval start; KO, knockout rounds/heats; M, men; M + W, mixed‐sex event; M&W, separate men's and women's events; MS, mass start; P, prone; PS, pursuit start; Q, qualification; QF, quarterfinal; S, standing; SB, shooting bout; SB/leg, shooting bouts per leg; SF, semifinal; TT, time trial; W, women.

^a^ Sex gap reported as an estimated percentage difference in speed (rather than time). For mixed events, M and W denote typical men's‐leg versus women's‐leg values, not separate men's and women's competitions.

^b^ Ski mountaineering: competition times and the magnitude of sex differences depend on course features.

*Source:* International Ski and Snowboard Federation (FIS) cross‐country competition rules; International Biathlon Union (IBU) event rules; International Ski Mountaineering Federation (ISMF) competition rules; IOC Milano–Cortina 2026 sports and event‐program documents; official competition results (FIS, IBU, ISMF); and peer‐reviewed analyses of performance in Winter Olympic snow sports.

#### 3.1.1 Cross‐Country Skiing

In the Milano Cortina 2026 Olympic cross‐country skiing program, skiers compete in three distance events—the 10‐km individual time trial, the 20‐km skiathlon, and the 50‐km mass start—alongside the 4 × 7.5‐km relay. Sprint competitions (1.2 km at the Milano Cortina Games) are contested in two formats: an individual event comprising a qualifying time trial and three knockout heats, and a team sprint consisting of semi‐final and final rounds in which pairs alternate three 1.2‐km legs. The race courses typically comprise approximately one‐third uphill, one‐third flat, and one‐third downhill terrain, resulting in ~50% of the total time spent racing uphill [10, 11].

This requires skiers to master many different sub‐techniques and efficient transitions between them at a wide range of speeds (5–70 km h^−1^) and on terrains with inclines varying from −20% to +20%. At present, 10 of the 12 Olympic events involve mass starts, where tactics are crucial for drafting to reduce air resistance, avoiding collisions and falls, skiing efficiently within a pack, and positioning for the final sprint, which often determines the outcome [12].

Across race formats, aerobic metabolism contributes approximately 70%–80% of total energy in sprint events and 85%–95% in distance races [13]. Anaerobic metabolism accounts for the remainder in response to variable terrain, fluctuating workloads, and frequent transitions between sub‐techniques that engage upper‐ and lower‐body musculature [10, 11, 13]. The application of higher intensity on uphill terrain drives work rates considerably above those required to elicit maximal oxygen uptake (VO_2_max) and requires an additional anaerobic component. Consequently, cross‐country skiing imposes exceptional physiological challenges, characterized by high aerobic and anaerobic energy turnover, efficient skiing technique, high‐speed ability, and well‐developed fatigue resistance [10, 11, 13]. Uniquely, world‐class cross‐country skiers exhibit some of the highest VO_2_max values ever reported in sport, with values of 80–90 and 70–80 mL kg^−1^ min^−1^ commonly observed in men and women, respectively, and absolute values for male and female medal winners exceeding 6.5 and 4.5 L min^−1^, respectively [14].

Sex‐based performance differences in cross‐country skiing are well documented. Competition speeds differ by 7%–16% between female and male skiers on comparable courses [15, 16, 17], with the primary determinant being men's 10%–15% higher relative V̇O_2_peak [10, 11, 17]. This physiological advantage is most apparent on uphill sections, where the high metabolic cost of working against gravity widens speed gaps to 18%–19% [11, 15, 18]. In addition, men exhibit greater absolute anaerobic capacity [19, 20], attributed to larger active muscle mass and higher glycolytic enzyme activity; this difference yields moderate‐to‐large effect sizes [17, 19, 20].

Cross‐country skiers continuously change between and adapt the sub‐techniques of classical skiing (diagonal stride, double poling with a kick, double poling, and herringbone) and ski skating (Gears 2, 3, 4, and 5) to the varying terrain [21]. Biomechanically, the fastest skiers achieve longer cycle lengths at high speeds on flat terrain, whereas maintaining rapid cycles while minimizing the reduction in cycle length is essential for accelerating on steep hills, during the start, and in the final sprint. The sex difference is most pronounced in techniques with a high upper‐body power contribution, such as double poling (~20%), compared with whole‐body techniques like diagonal stride (~14%) [11, 22, 23]. Men's superior propulsive capacity enables longer cycle lengths and the utilization of higher “gears” (sub‐techniques) across varying terrains and intensities [15, 16, 18, 20, 24]. Consequently, technique distribution differs substantially between sexes: in classic races, women spend significantly more time using diagonal stride and less time double poling than men [16, 18]. This difference in technique utilization contributes to the distinct race characteristics observed in men's and women's Olympic cross‐country events. To objectively quantify these technical demands, high‐precision GNSS technology is increasingly employed to analyze trajectory choices and speed profiles, providing granular insights into technique distribution across varying topographies (see [7]).

The stochastic interval profile of cross‐country skiing, combined with the continuous switching between sub‐techniques, necessitates a high ability to self‐regulate effort during both training and competition. Consequently, the concept of pacing has been extensively studied over the last decade (see [5]) and is strategically implemented by elite skiers to optimize performance [25]. Optimizing pacing strategies and tactical decision‐making requires specific, repeated training that should be integrated from an early stage of a skier's career [25].

Despite these performance disparities, the underlying training principles and volumes are largely similar between elite male and female cross‐country skiers. Annual training volumes typically reach ~750–1050 h, with a polarized or pyramidal intensity distribution dominated by low‐intensity training (LIT; ~80%–90%), supplemented by 2–3 weekly sessions of moderate‐ and high‐intensity work and year‐round strength training emphasizing upper‐body power and muscular endurance [11, 26, 27, 28]. Preparation often includes altitude exposure (see [2, 3]), with athletes either residing at moderate elevation (~1800–2500 m) or completing multiple altitude camps totaling 60–100 days per year [11, 27, 29]. In some camps, athletes may reside at ~1200–2000 m while completing on‐snow ski training on nearby glaciers at ~2700–3000 m (e.g., Ramsau; Val Senales). Furthermore, the annual macrocycle typically progresses from a preparation period with extensive low‐intensity training to a competition season, characterized by increased high‐intensity training and numerous races, a periodization pattern that appears beneficial for the long‐term development of elite endurance athletes. At the same time, observations indicate that the very best athletes competing in the Olympics are increasingly focusing on the quality of each session (i.e., optimization of physical, technical, and mental aspects) rather than the volume alone [30, 31].

Despite this extensive evidence base, critical knowledge gaps persist. These include optimizing long‐term athlete development pathways, understanding individual variability in training responses and competition strategies, and refining sex‐specific training prescriptions. Moreover, the integration of on‐snow monitoring technologies and the influence of environmental factors (e.g., snow conditions, ski–snow interaction) on performance and technique warrant further investigation.

#### 3.1.2 Biathlon

Biathlon is a bimodal sport that combines high‐intensity ski skating with high‐precision rifle shooting [32, 33]. Unlike most other endurance sports, performance is determined not only by movement speed but also by the execution of a fine‐tuned motor task. Overall performance can be partitioned into three components: skiing time (course time), range‐ and shooting time, and number of shooting hits (penalty time). The relative impact of these factors varies by race format. In sprint races (7.5 km for women and 10 km for men), skiing time explains ~60%–65% of the variance in overall performance, whereas penalty loop time (typically 22–25 s) accounts for ~30%–35% [34]. In contrast, in the individual race (15 km for women and 20 km for men), where each missed shot results in a fixed 1‐min time penalty, the contribution of penalty time equals or exceeds that of skiing time [35, 36]. Notably, among elite biathletes of both sexes, shooting performance (number of hits)—rather than skiing speed—is the most decisive factor distinguishing podium finishers from other competitors [36].

The physiological demands of biathlon skiing are similar to those of cross‐country skiing, with frequent variations in speed and terrain requiring highly developed maximal aerobic power, the ability to restore anaerobic capacity during downhill sections, and technical efficiency across various sub‐techniques (gears). However, a key distinction is skiing with a rifle, which increases physiological demands compared with standard cross‐country skiing. The identical rifle mass requirement (≥ 3.5 kg) imposes a relatively greater load on women [33]. Rifle carriage elevates oxygen cost and metabolic rate at fixed speeds, whereas gross efficiency remains unchanged [37], yet it modifies biomechanics and sub‐technique selection [37, 38], with the greatest effects occurring in uphill terrain and during transitions [38]. Together, these physiological and biomechanical differences result in faster skiing speeds among men, accounting for more than 90% of the sex difference in total race time; on equivalent courses, women's skiing times are ~12%–15% longer [34, 35]. Although men also demonstrate marginally faster shooting and range times, these factors contribute only modestly (~7%–8%) to the overall sex‐based performance difference [34, 35].

The combination of high‐intensity skiing across various disciplines with rifle marksmanship offers an intriguing context to study, develop, and optimize pacing strategies. In general, pacing behavior, overall effort, and the use of perceptual information to adjust speed in biathlon are similar to cross‐country skiing [25]. However, shooting requires biathletes to reduce speed substantially in the last ~60 s approaching the range, likely to facilitate breathing stabilization, a key factor in shooting performance. Consequently, similar to cross‐country skiing, developing an athlete's ability to self‐regulate effort appears to be a crucial performance characteristic for reaching an elite level [39, 40].

To meet biathlon's dual physiological and technical demands, elite athletes perform approximately 700–900 h of physical training annually. Low‐intensity training (LIT) constitutes ~80% of this physical training total volume, supplemented by 2–3 weekly moderate‐ and high‐intensity sessions (MIT/HIT). In addition, many biathletes incorporate regular altitude training camps [32, 41]. This endurance framework is systematically integrated with rifle marksmanship; world‐class biathletes fire 20 000–22 000 shots annually, approximately 60% of which are executed during endurance sessions to simulate shooting under elevated physiological stress [32]. To address the complex psychophysiological demands of shooting under load, athletes also employ cognitive techniques; for instance, structured programs incorporate autogenic and imagery training to improve standing shooting performance by enhancing postural control and holding ability [33]. Longitudinal data suggest that elite athletes may progressively transition toward more quality‐oriented, lactate‐guided models; for instance, a case study of a world‐class female biathlete linked peak performance to increased MIT and greater precision/dry‐fire volume, even with slightly reduced total annual hours during the senior phase [41].

#### 3.1.3 Ski Mountaineering

Uphill performance in ski mountaineering—a newly included Olympic sport—is the primary determinant of finishing time in both sprint and mixed relay formats, accounting for ~80%–90% of performance variance [42, 43]. Transition efficiency, defined as the rapid execution of equipment changes (e.g., skin attachment/removal, switching between skiing and boot‐packing), explains a further ~10%–15% of variance and significantly differentiates podium‐level athletes from mid‐field competitors [42, 43].

The sprint discipline comprises a single ascent and descent (~70 m elevation gain/loss) punctuated by three technical transitions (i.e., skin‐to‐boot, boot‐to‐skin, and skin‐to‐ski). The competition unfolds over multiple rounds, progressing from an individual time‐trial qualification to knock‐out heats of six athletes. In contrast, the mixed relay is contested in a circuit featuring two ascents and descents (120–150 m elevation gain/loss) and six complex transitions. The competition is contested by mixed teams composed of one male and one female athlete. Each athlete completes the circuit once during the time‐trial qualifications, with the team's result determined by the aggregated time, and twice in the head‐to‐head final, in the order woman–man–woman–man.

The short, intermittent nature of these events—with efforts lasting approximately 2.5–3.5 min in sprints and ~7–10 min in mixed relays, combined with partial recovery during downhills—imposes considerable demands on both aerobic and anaerobic energy systems. While laboratory‐derived maximal aerobic indices (e.g., vertical speed at V̇O_2_max) best predict individual performance in the mixed relay, supramaximal aerobic‐anaerobic indices (e.g., 2‐min mean vertical speed) are stronger predictors of sprint outcomes [43].

Inter‐athlete variability in uphill performance is driven by more than metabolic power alone; given the sport's technical demands, ascent speed is heavily influenced by neuromuscular, anthropometric, and biomechanical factors that shape movement efficiency [44, 45]. For instance, lower lean‐to‐fat mass ratios, combined with lower absolute strength, may underlie the greater vertical energy cost and reduced efficiency observed in women during sub‐maximal aerobic exercise, where sex‐related performance gaps are typically ~15%–20% [43, 46]. This sex disparity widens to ~25%–30% during the short, supramaximal efforts characterizing the sprint format, which relies more heavily on anaerobic metabolism (~35%–40% of total energy over a 2‐min effort) and muscular power [43].

To date, data regarding the anthropometric characteristics of Olympic‐format ski mountaineers remain limited. In the more extensively studied vertical, individual, and long‐distance disciplines, performance time is negatively associated with fat mass, body fat percentage, and BMI, as excess adiposity impairs the critical power‐to‐weight ratio and is linked to slower uphill performance outcomes [47, 48, 49]. Conversely, in the sprint discipline, a distinct anthropometric profile—characterized by higher fat‐free mass and BMI alongside low fat mass—appears to be associated with superior performance, at least among male athletes [43, 50].

Elite ski mountaineers accumulate annual training volumes of ~650–950 h, underpinned by a robust aerobic base and multimodal conditioning. Ski mountaineering comprises approximately 45% of total volume, complemented by running, cycling, roller‐skiing, and strength training [51]. Altitude camps are frequently utilized to extend on‐snow preparation and acclimatize to hypoxic competition conditions. Training programs target the ability to repeat near‐maximal efforts and supramaximal efforts, requiring the development of high maximal aerobic power (peak V̇O_2_ > 70 mL kg^−1^ min^−1^ in women; > 80 mL kg^−1^ min^−1^ in men), superior movement efficiency, an optimized power‐to‐mass ratio, and substantial anaerobic capacity [42, 43]. Critical technical focuses include optimizing transition proficiency and improving performance repeatability for multi‐round competitions [43]. While these principles apply broadly, national team data indicate that female athletes typically complete 15%–20% lower annual volumes than men [51]. Future research should investigate how periodization strategies—specifically the balance of endurance volume, high‐intensity anaerobic training, and altitude exposure—can best optimize physiological adaptations for these specific Olympic disciplines.

### 3.2 Gravity and Technical Sports

This category encompasses disciplines characterized by high‐speed descents, strength‐ and power‐dependent actions, high‐coordination tasks or tricks, and the capacity to maintain precise motor control under high external forces and variable environmental exposure. Performance is influenced by neuromuscular strength, rate of force development, aerodynamic optimization, ski–snow interaction, and equipment setup, as well as perceptual‐motor‐cognitive function, tactical decision‐making, and the ability to manage perturbations and risk. Consequently, beyond the complex physical demands, these sports also require superior mental skills for optimal performance. While the scientific characterization of these complex demands is less extensive than in endurance sports, current competition data (Table 2) quantify elite‐level benchmarks regarding speed, technical difficulty, and aerial complexity.

**TABLE 2:** Overview of event characteristics, format regulations, and performance metrics for gravity, technical and hybrid snow sports at the Milano–Cortina 2026 Olympic Winter Games.

Sport	Event	Category	Format	Performance metrics	M	W	Sex gap (%)
Alpine Skiing (10)	Slalom	M&W	Two runs	Mean speed (km h^−1^)^a^	≈45–48	≈41–44	≈3%–4%
	Giant Slalom	M&W	Two runs	Mean speed (km h^−1^)^a^	≈60–65	≈56–60	≈3%–4%
	Super‐G	M&W	Single run	Top speed (km h^−1^)^a^	≈120–135	≈110–125	≈3%–4%
	Downhill	M&W	Single run	Top speed (km h^−1^)^a^	≈135–155	≈130–140	≈3%–4%
	Team Combined	M&W	Teams of 2, 1 DH and 1 SL run		—	—	—
Freestyle Skiing (15)	Aerials	M&W	Multiple judged jumps; Q + Final	DD/score (pts)	4.3–5/≈120	3.3–4.2/≈96	≈15%–20%
	Mixed‐Team Aerials	M + W	Team of 3; combined score		—	—	—
	Moguls	M&W	Multiple runs; Q + Final	Time (s)/score (pts)	≈21–23/83–89	≈23–25/80–86	≈10%–14%/3–5 pts
	Dual Moguls	M&W	Head‐to‐head rounds	Time (s)/score (pts)	≈21–23/83–89	≈23–25/80–86	≈10%–14%/3–5 pts
	Halfpipe	M&W	Multiple runs; Q + Final	Max rotation (°)^b^	≈1620	≈1260	—
	Slopestyle	M&W	Multiple runs; Q + Final	Max rotation (°)^b^	≈1980	≈1620	—
	Big Air	M&W	Multiple judged jumps; Q + Final	Max rotation (°)^b^	≈2160	≈1620	—
	Ski Cross	M&W	Multiple runs; Q + KO rounds	Time (s)	≈45–80	≈50–85	≈5%–6%
Snowboarding (11)	Parallel Giant Slalom	M&W	Head‐to‐head runs; Q + KO rounds	Time (s)	≈60–85	≈65–90	≈5%–6%
	Snowboard Cross	M&W	Multiple runs; Q + KO rounds	Time (s)	≈40–55	≈45–60	≈8%–10%
	Halfpipe	M&W	Multiple judged runs; Q + Final	Max rotation (°)^b^	≈1440	≈1080	—
	Slopestyle	M&W	Multiple judged runs; Q + Final	Max rotation (°)^b^	≈1620	≈1260	—
	Big Air	M&W	Multiple judged jumps; Q + Final	Max rotation (°)^b^	≈1980	≈1440	—
	Mixed‐Team Snowboard Cross	M + W	W–M; combined time	Time (s)	≈40–55	≈45–60	≈8%–10%
Ski Jumping (6)	Individual Normal Hill	M&W	NH (HS 109 m); 2 jumps	Points^c^	—	—	≈8%–14%
	Individual Large Hill	M&W	LH (HS 142 m); 2 jumps	Points^c^	—	—	≈14%–24%
	Super Team Large Hill	M	LH, 2 jumpers; 3 rounds; team score	Points^c^	—	—	—
	Mixed Team Normal Hill	M + W	NH, 4 jumpers; 2 rounds; team score	Points^c^	—	—	≈8%–14%
Nordic Combined (3)	Individual Normal Hill/10 km	M	1 NH jump; 10 km XC (F), (Gundersen)	Time (min)^d^	≈25–30	—	N/A
	Individual Large Hill/10 km	M	1 LH jump; 10 km XC (F), (Gundersen)	Time (min)^d^	≈25–30	—	N/A
	Team Sprint	M	Teams of 2; 1 jump/ath; 2 × 7.5 km XC (F)	Time (min)^d^	≈30–40	—	N/A

*Note:* Number of medal events in parentheses. Sex Gap (%): Estimated percentage difference between men and women in the reported performance metric (time or speed). Combined time/score metrics: Sex Gap values are reported as time difference (%)/score difference (pts). In Gundersen‐format events, ski‐jumping points are converted to time handicaps for the XC start (commonly 1 pt = 4 s in individual events; 1 pt = 1.33 s in the team sprint).

Abbreviations: DD, degree of difficulty; DH, downhill; F, free technique (XC) (used only where XC technique is specified, e.g., Nordic combined); HS, hill size; KO, knockout rounds; LH, large hill; M, men; M + W, mixed‐sex event; M&W, separate men's and women's events; NH, normal hill; Q, qualification; SL, slalom; W, women; XC, cross‐country skiing.

^a^ Alpine skiing: Speeds are strongly influenced by course setting, snow conditions, and venue topography.

^b^ Freestyle skiing & snowboarding: Rotation values (degrees) reflect current elite‐level progression; “Max rotation (°)” denotes the typical maximum landed rotation; maximum landed rotations may increase by the 2026 Games.

^c^ Ski jumping: Point‐based score derived from distance and style, with wind and gate compensation.

^d^ Nordic combined: Hybrid sport (ski jumping + XC). At Milano–Cortina 2026, Nordic combined remains a men's‐only Olympic discipline (three men's events).

*Source:* International Ski and Snowboard Federation (FIS) competition rules and course specifications (alpine skiing, freestyle skiing, snowboarding, ski jumping, and Nordic combined); IOC Milano–Cortina 2026 event program documents; official competition results (FIS); and peer‐reviewed analyses of performance in winter Olympic snow sports.

Across gravity and technical sports, distinct physiological and biomechanical demands intersect to form three functional archetypes: (1) power‐ and gravity‐assisted sports (e.g., alpine speed events such as downhill and super‐G, and snowboard cross), where higher total mass coupled with substantial neuromuscular power enhances start performance and momentum generation; (2) technical, turn‐intensive disciplines (e.g., alpine slalom and giant slalom, snowboard parallel events, ski cross, and moguls), which favor low rotational inertia to enable rapid edge transitions, precise timing, and effective re‐acceleration; and (3) flight‐ and rotation‐dominant sports (e.g., ski jumping, freestyle aerials, and snowboard big air and slopestyle), which benefit from favorable height‐to‐mass ratios that optimize take‐off mechanics, angular‐momentum control and aerial maneuverability. While these archetypes highlight shared performance determinants, the training cultures and scientific literature are organized by specific disciplines. Consequently, the following sections review the evidence by sport.

#### 3.2.1 Alpine Skiing

Competitive alpine skiing comprises four basic disciplines—slalom (SL), giant slalom (GS), super‐G (SG), and downhill (DH)—that differ in duration, speed, and degree of directional change, thereby imposing diverse demands on athletes [52, 53]. These differences are shaped primarily by terrain characteristics and course setting (e.g., gate distance and horizontal offset), which collectively determine average and peak skiing speeds [54]. Success at the elite level depends on highly developed technical and tactical skills, supported by psychological attributes (e.g., mental toughness, fear management, and emotion regulation) and physical capacities such as muscular strength and power, aerobic and anaerobic endurance, balance, core stability, and coordination [53, 55, 56, 57, 58, 59, 60, 61]. However, athletes with a wide range of anthropometric and physiological profiles can succeed at the top level [53, 62].

A critical biomechanical constraint involves managing high ground‐reaction forces (GRFs). In SL, peak GRFs typically reach ~4× body weight (BW) [63, 64], with occasional peaks approaching ~5× BW [65], whereas in GS and SG/DH peak forces are lower at ~3.2× and ~2.6× BW, respectively [53, 66, 67]. However, it is important to note that these values represent filtered peak values observed during controlled skiing. These values may be even higher in the form of low‐frequency vibrations while turning, jump landings, or in the event of an injury [68, 69, 70].

For comparison, level running elicits vertical GRFs of ~2–3× BW, rising to ~3.5–4× BW during sprinting [71]. However, unlike running, where GRF_S_ occur during brief contact times of < 0.10–0.20 s depending on speed [71, 72], ski turn forces are sustained throughout the steering phase, which typically lasts 0.40–1.20 s depending on the discipline [63, 66]. This prolonged loading phase requires skiers to generate distinct muscular adaptations—specifically, the capacity to tolerate high force‐time integrals (impulse) rather than purely reactive, plyometric impacts. The effective management of such high relative GRFs underscores the critical role of technique and neuromuscular control in maximizing performance across sexes, despite potential differences in absolute force production [73].

A further determinant of performance is minimizing energy dissipation, primarily from ski‐snow friction and aerodynamic drag [74, 75, 76]. While this is especially critical in the speed events of SG and DH [52], performance in the technical disciplines of SL and GS is more complex [67, 77]. In SL and GS, the fastest times result not only from minimizing energy loss but also from optimizing line choice and timing—navigating trade‐offs between tight versus rounder turns to maintain speed while managing forces and fatigue [67, 77, 78]. Thus, the interplay between physical capacity and technical execution is fundamental to success in all disciplines [52, 62].

The training regimen for elite alpine skiers is complex and periodized, targeting multiple physical capacities concurrently [53]. An athlete's specialization—typically in either technical (SL, GS) or speed (SG, DH) events—guides the distribution of training volume, although the total number of on‐snow training days (typically 100–150 per year) remains similar [53]. While men and women compete on terrain of comparable technical characteristics (e.g., general steepness and snow conditions), FIS regulations define discipline‐ and sex‐specific course specifications—most notably prescribed vertical drop. Consequently, women's courses are generally shorter and sometimes utilize lower start positions and/or minor layout adjustments compared with men's events, likely reflecting sex‐based differences in absolute power and resulting on‐snow performance. On‐snow sessions consist of partial‐ to full‐length runs on competition‐simulated courses, with run counts scaled to event demands (e.g., 6–12 runs per session for SL, 4–8 runs per session for DH). The ceiling for on‐snow training volume is determined less by fatigue than by specificity: at sub‐race speeds, ski–snow interaction often fails to generate the bending and edge forces required to reproduce race‐specific technique and timing; consequently, additional low‐speed runs yield limited transfer but remain important for expanding the range of technical skills. To objectively quantify these technical demands, on‐slope data collection—particularly GNSS‐based trajectory tracking and emerging force‐sensing technologies—is becoming a critical tool for performance analysis, although its integration into routine daily training is still evolving.

This on‐snow work is supported by extensive off‐snow conditioning, typically totaling 14–21 h week^−1^ distributed across 10–14 sessions [53], which targets the whole body, with special emphasis on the legs, core, and hip/gluteal region [53, 61]. Programs integrate endurance (e.g., cycling and running), various forms of strength (maximal, explosive/plyometric, and strength endurance), agility/motor control, coordination and stability. Injury prevention is also integrated—a critical focus given the high prevalence of knee injuries (e.g., ACL tears) in female skiers—particularly targeting lower‐limb alignment by addressing the hip external rotators, maximal eccentric hamstring strength, and core stability [53, 56, 79]. While the overall conditioning approach is similar, speed specialists place greater emphasis on endurance and strength, whereas technical skiers prioritize quickness and power [53]. Although reported training volumes are not stratified by sex, the underlying principles and loading strategies appear broadly representative of both women and men.

#### 3.2.2 Freestyle Skiing

Freestyle skiing comprises multiple disciplines—including Aerials (AE), Moguls (MO), Ski cross (SX), and Park & Pipe (P&P)—each with distinct performance determinants, ranging from head‐to‐head racing in SX to judged execution in AE, MO, and P&P. Judgment criteria vary by discipline: AE evaluates air (20%), form (50%), and landing (30%); MO emphasizes turns (60%), air (20%), and speed (20%); and P&P assesses execution, difficulty, amplitude, variety, and progression [80]. In SX, performance is determined purely by ranking at the finish line, with start acceleration being crucial for initial positioning [81].

The demands of AE are unique, combining acrobatic gymnastics with ski‐jumping mechanics. Athletes descend at ~50–70 km h^−1^ and launch from a 6–8 m kicker, achieving vertical drops of up to ~18 m, during which they execute complex aerial maneuvers, typically involving multiple somersaults and twists. At the Olympic level, male competitive routines frequently demand triple somersaults incorporating 4–5 twists. In contrast, elite female athletes typically perform double somersaults with up to 3 twists, with podium performances increasingly requiring triple somersaults involving 1–2 twists [82, 83]. Airtime is governed by launch height and the vertical component of take‐off velocity—where approach speed is redirected vertically at the kicker. Simultaneously, the generation of angular momentum (via angular impulse) at take‐off is critical, as it dictates the capacity for complex aerial maneuvers. This rotation is initiated through asymmetric force application and rapid segmental movements of the lower limbs and trunk. Subsequently, in‐flight tilt, generated by asymmetrical arm and hip actions, modulates the twist rate and its cessation [82, 83, 84]. Recent performance modeling identified training experience, relative maximum anaerobic power, maximum lower‐limb strength, and core stability as the strongest predictors for the top AE performers [82]. Sex‐related differences in factors such as lower‐body power likely contribute to variations in flight amplitude and achievable trick complexity [82, 85].

Technical and acrobatic intricacies also characterize the other judged disciplines. In MO, success is driven by high‐speed skiing over densely spaced moguls on a steep (up to 32°) slope, requiring well‐timed absorption–extension strategies to manage vertical forces, a stable upper body, and clean aerial maneuvers [86, 87]. Acrobatic skills underpin performance in the three P&P disciplines—Halfpipe (HP), Slopestyle (SS), and Big Air (BA). Across these formats, performance is determined by the execution and difficulty of aerial maneuvers [61]. In HP, executing complex rotations relies on generating sufficient angular momentum at take‐off, achieving sufficient airtime, and maintaining control throughout aerial maneuvers [61]. In SS, performance is characterized by fluid linking of jumps and rails, managing landing (impact) forces of ≈2× BW, and adapting to variable course designs and feature sequences [61, 88]. In BA, which consists of a single jump unlike other Park & Pipe disciplines, success is driven primarily by trick difficulty. This, in turn, requires optimized take‐offs for angular momentum generation, extended airtime, and controlled landings to meet execution standards [61, 89].

In contrast to these judged events, SX is strictly race‐oriented. Performance hinges on an effective start: athletes must generate high initial velocity and resultant impulse at the start gate [81]. Thereafter, success depends on the ability to efficiently negotiate course features—such as rollers, jumps, and banked turns—at high speed, with technical proficiency influencing speed maintenance, tactical overtaking, and the use of slipstreaming, which can aid in closing gaps and gaining position, ultimately determining race outcome [90].

Although physical capacities such as lower‐limb power, core stability, and aerial coordination underpin performance—and exhibit sex‐related variation—preparation in freestyle skiing generally follows a consistent technical development model across sexes. This model integrates foundational strength and neuromuscular training with a staged skill acquisition sequence (trampoline → water‐ramp/airbag → snow) [61, 91, 92]. Off‐snow conditioning targets event‐specific capacities: P&P athletes emphasize take‐off power, rotational control, and landing mechanics; AE athletes benefit from trunk‐stability interventions, such as an 8‐week core program shown to improve landing‐phase kinetics and trunk control [93]. MO preparation focuses on eccentric strength and vertical agility for rapid absorption and turn execution [87], whereas SX emphasizes explosive start performance and whole‐body coordination using custom ramps replicating race conditions [81]. Across all disciplines, psychological preparedness is critical. Like many snow sports, freestyle skiing requires athletes to adapt continuously to environmental unpredictability, demanding refined cognitive skills and robust coping strategies to manage risk and performance under pressure [58, 61].

Notably, most current biomechanical and training research in freestyle skiing and snowboarding derives from male or mixed‐sex cohorts. There is a pressing need for studies focused specifically on elite female athletes to ensure equitable and evidence‐based preparation strategies moving forward.

#### 3.2.3 Snowboarding

In the Milano Cortina 2026 program, snowboarding encompasses a diverse range of competitive disciplines, broadly divided into judged events (Park & Pipe: Halfpipe, Slopestyle, Big Air) and raced events (Parallel Giant Slalom [PGS] and Snowboard Cross [SBX]) [94].

The judged disciplines entail either the execution of a single maneuver (Big Air) or a sequence of tricks (Halfpipe or Slopestyle). In both formats, performance hinges on five primary criteria: execution, difficulty, amplitude, variety, and progression [80]. The weighting of these factors reflects discipline‐specific demands; for example, amplitude and rotation complexity are prioritized in Big Air (BA) and Halfpipe (HP), whereas rail execution and flow carry greater weight in Slopestyle (SS). Current progression benchmarks highlight clear sex‐specific performance baselines: in Big Air, top male athletes currently execute quadruple corks with 1800°–2160° of rotation (5–6 revolutions), whereas top female athletes typically perform double or triple corks with 1260°–1440° of rotation (3.5–4 revolutions).

To maximize scoring potential, athletes utilize aerodynamic postures and apparel to reduce in‐run drag—driven primarily by posture and to a lesser extent by apparel [95]—thereby increasing take‐off speed and available airtime. Landing stability is influenced by equivalent fall height and maneuver complexity (e.g., angular velocity, axis of rotation); notably, snowboarders are generally less stable than skiers on comparable rollover jumps [96]. Furthermore, compared with timed events, judged events typically exhibit greater within‐athlete performance variability and lower predictability, underscoring the importance of execution consistency [97].

In contrast, performance in the raced disciplines is determined strictly by elapsed time. Success in PGS and SBX relies on explosive start performance (especially crucial in SBX) and the ability to sustain high‐force carving throughout the course; in addition, SBX demands precise timing of energy‐pumping movements over rollers and technical precision on jumps (e.g., absorbing take‐offs to maintain low, fast trajectories) [98, 99, 100].

Despite these structural differences, research reveals that key physical determinants—especially strength and power—are critical to performance across all disciplines [94]. In raced events (PGS and SBX), faster times are associated with higher maximal isometric strength, power output at ventilatory thresholds, peak power output (absolute/relative), and leg stiffness [98, 99]. Similarly, for judged events such as HP, lower‐body power supports greater amplitude, which provides the necessary airtime for complex tricks [101]. Recent analyses indicate that sex accounts for more variance in physiological test results than discipline specialization does, with males generally exhibiting greater jump height, absolute/relative strength, and aerobic capacity [85, 98]. While such physiological differences influence performance, current evidence supports a broadly similar training methodology across sexes, applied with athlete‐centered individualization [61, 85].

Preparation for elite snowboarding integrates on‐snow technical practice with targeted off‐snow conditioning following a “test → train → on‐snow integration” cycle guided by regular profiling and goal‐directed adjustment [61]. Training is periodized around the season schedule: off‐season training focuses on strength/power development and acrobatic training, whereas in‐season training focuses on skill maintenance and recovery between events. However, specific content diverges by discipline: Park & Pipe athletes focus on developing amplitude‐generating power, reactive jump capacity, aerial coordination, and landing control. The latter—particularly stiffness modulation—is developed through plyometrics and progressive training environments such as trampolines and airbags [61, 96, 101, 102]. Conversely, PGS and SBX racers prioritize maximal and explosive lower‐limb strength for powerful starts and high turn forces; maintaining these forces across the course benefits from well‐regulated leg stiffness, with aerobic capacity assisting run duration and between‐heat recovery in SBX [94, 98, 99].

A knowledge gap remains in identifying the optimal training periodization and monitoring for snowboarders. The “test–train–retest” approach is advocated, but consensus on standardized testing protocols for talent development is lacking. While very little information is generally available on snowboard physiology and training, most performance data (e.g., strength and power) come from male athletes. Research on elite female snowboarders' physiology is non‐existent, although current training evidence suggests that they benefit from the same training emphasis as men do when training is individualized.

#### 3.2.4 Ski Jumping

Competitive success in ski jumping is quantified by a point‐based metric derived 40%–60% from physical jump length and 40%–50% from style, with the remaining 10%–20% attributed to compensation points to ensure fairness in variable external conditions [103]. The main focus is on the physical performance of the take‐off, recognized as the most important phase [104, 105, 106, 107]. Here, the athlete aims to create as much vertical force as possible and sufficient rotation. This, together with maximizing in‐run speed, sets the initial conditions for the flight, where the aim is to maximize horizontal speed while minimizing vertical speed [108]. The importance of aerodynamic efficiency has intensified over the last century, where the world record has increased by ~1.7 m year^−1^, making the aerial phase increasingly critical [109]. Consequently, how post take‐off parameters influence performance has been investigated using both computer simulations [110] and field investigations [111, 112]. Elfmark et al. [108] measured the aerial phase with dGNSS and found that performance during this phase was highly important in large hills. Specifically, a low angle of attack (body angle to the trajectory) during the flight was found to significantly influence performance, in contrast to the normal hill, where performance was influenced mainly by in‐run speed and take‐off parameters [108].

Equipment represents a critical performance variable in modern sports, and ski jumping is no exception. While strict regulations govern the design of skis, bindings, and boots to ensure fairness and safety, numerous disqualifications occur each year, the majority resulting from non‐compliant suits. This issue culminated at the 2025 World Championships, where five athletes were suspended [113]. The performance impact of such equipment was highlighted by Elfmark et al. [103], who reported a 16% improvement in men's performance from training to competition, presumably due to the use of optimized competition equipment (e.g., new suits) compared to worn training gear. However, research in this area remains limited, as stakeholders are often reluctant to participate in investigations involving sensitive equipment. Recently, the FIS has initiated targeted efforts. For example, Virmavirta et al. [113] used wind tunnel measurements and simulations to demonstrate that increasing suit circumference by 1 cm could increase jump length by 2.8 m.

Due to the nature of the sport, a ski jumper is restricted to a limited number of jumps per training session. To supplement this limited on‐hill air time, some athletes have begun exploring new pre‐season training methods, such as inclined wind tunnel training, to enhance motor learning and flight technique. In one of the few studies including female athletes, Elfmark et al. [114] demonstrated the importance of practical experience on appropriate hill sizes, as women improved markedly during the first‐ever female ski flying competition. For daily training, imitation jumps, designed to mimic the take‐off movement, are commonly utilized and have been the focus of several studies [115, 116, 117]. Imitation jumps achieve the highest ecological validity when a rolling platform and, preferably, ski jumping boots are used [116]. In addition, ski jumpers require significant strength, as they experience transient peak ground reaction forces of up to 5× BW on the lead leg upon landing. The objective here is twofold: to ensure injury prevention and enable a controlled telemark landing for maximum style points [118]. Finally, while a low body weight is beneficial for performance, it influences both training protocols and general health [105]. However, how this affects training methods in ski jumping remains poorly understood.

Despite its long‐standing Olympic status, ski jumping research remains limited in several areas. The main limitation is the focus on women, who have been mentioned in the ski jumping literature only sporadically [114]. Other topics with limited research include the optimization of individualized take‐off mechanics, the influence of equipment, the impact of environmental conditions (such as wind variability), and the development of sex‐specific training and health monitoring protocols. Addressing these gaps is essential for advancing performance and safeguarding athlete well‐being, particularly as the sport continues to evolve in the lead‐up to Milano Cortina 2026 and beyond.

### 3.3 Nordic Combined: A Hybrid Discipline

Nordic combined (NC) uniquely merges ski jumping and cross‐country skiing into a single competition format, typically comprising a ski jump followed by a 10‐km skating‐style pursuit race. Overall performance is determined by a combination of jump score and skiing time, with the Gundersen method used to convert jumping results into staggered start times for the cross‐country race [17, 119]. This dual‐discipline format imposes contrasting physiological and technical demands. Ski jumping requires explosive lower‐body power, precise timing, and aerodynamic efficiency, whereas cross‐country skiing demands high aerobic capacity, movement economy, and fatigue resistance [117]. NC athletes must therefore develop both maximal power and endurance capacities while managing the interference effects that can arise from concurrent training [120].

Despite performing only ~50%–60% of the discipline‐specific training volume of specialists in each sport, NC athletes exhibit only ~10%–17% lower laboratory‐measured capacities than elite ski jumpers and cross‐country skiers [117, 121]. This suggests that well‐structured, long‐term development programs can effectively integrate the divergent demands of NC. Training is typically periodized to balance the development of both components. Athletes alternate between blocks emphasizing ski jumping technique and power, and blocks focused on endurance training, including roller skiing, running, and strength‐endurance work [121]. Strength training is tailored to support both explosive take‐off mechanics and cross‐country‐specific muscular endurance, with careful attention to body weight management to avoid compromising jump performance [122].

Women's Nordic combined has been included in the FIS World Cup since the 2020/2021 season but is not yet part of the Olympic program. As a result, scientific data on female NC athletes remain scarce. Given the physiological and technical challenges of combining two demanding disciplines, future research should explore sex‐specific training adaptations, optimal periodization strategies, and the long‐term development of female athletes in NC.

Additional knowledge gaps include the optimization of recovery strategies between the two events, typically held on the same day with a 1–3 h interval, and the identification of individual trade‐offs in training emphasis. For example, some athletes may benefit more from prioritizing ski jumping to secure a favorable start position, while others may rely on superior cross‐country capacity to compensate for lower jump scores. Understanding these individual profiles could allow for more personalized training approaches and talent development pathways.

## 4 Conclusions

This narrative review synthesizes key performance determinants and training practices across the snow‐based disciplines of the Milano Cortina 2026 Winter Olympic Games. These sports can be categorized into endurance‐dominant disciplines (cross‐country skiing, biathlon, ski mountaineering) and the gravity and technical disciplines (alpine skiing, freestyle skiing, snowboarding, and ski jumping), with Nordic combined requiring a complex integration of both. While endurance sports place primary emphasis on aerobic endurance training, complemented by sport‐specific strength and speed work, gravity and technical disciplines prioritize maximal strength and power, aerodynamic optimization, technical precision, tactical decision‐making, mental skills, and the management of high external forces through precise neuromuscular control. Current literature reflects a significant disparity: whereas cross‐country skiing, alpine skiing, and biathlon benefit from a more established evidence base, disciplines with a shorter Olympic history (e.g., freestyle skiing, snowboarding, and ski mountaineering) remain comparatively under‐researched. Moreover, the scarcity of data on elite female athletes across most disciplines continues to constrain the development of sex‐specific performance models.

## 5 Perspectives

To advance athlete development and optimize performance, future efforts should prioritize the integration of sport‐specific, individualized, and sex‐informed approaches. This includes leveraging emerging technologies for on‐snow monitoring, standardizing training documentation, and addressing critical gaps in injury prevention and energy availability, particularly in disciplines where power‐to‐weight ratios are decisive (see [4, 6]). By embracing multidisciplinary research and fostering collaboration between scientists, coaches, and athletes, the field can move toward more comprehensive, equitable, and effective performance frameworks. These efforts are essential to support success at Milano Cortina 2026 and will shape the future of Olympic snow sports.

## Author Contributions

C.Z., A.F., and H.‐C.H. conceived the review and planned and coordinated the literature search. C.Z., A.F., and H.‐C.H. drafted the initial manuscript. All authors contributed to interpretation of the findings, content development, and critical revision of the manuscript, and reviewed and approved the final version.

## Funding

The authors have nothing to report.

## Ethics Statement

The authors have nothing to report.

## Conflicts of Interest

The authors declare no conflicts of interest.

## Data Availability

All relevant data generated or analyzed during this study are included in this published article.
